# Neonatal Rectal Bleeding Due to Early‐Onset Ileocecal Intussusception: A Case Report

**DOI:** 10.1155/crpe/2932433

**Published:** 2026-06-18

**Authors:** Malaz Elnagi Musa Elshiekh, Maysoun Mahmoud Almusaddar, Farida Mohsin Sulaiman Ambusaidi

**Affiliations:** ^1^ Neonatal Intensive Care Department, Royal Hospital, Muscat, Oman, moh.gov.om; ^2^ Pediatric and Neonatology Department, Ibri Hospital, Ibri, Oman; ^3^ Radiology Department, Ibri Hospital, Ibri, Oman

**Keywords:** ileocecal intussusception, intussusception, necrotizing enterocolitis mimic, preterm neonate, rectal bleeding, ultrasonography

## Abstract

**Background:**

Intussusception is a well‐recognized cause of intestinal obstruction in infants but is rare in neonates, particularly in preterm infants. Because its clinical presentation can resemble necrotizing enterocolitis (NEC), diagnosis may be delayed when imaging findings are nonspecific. Early recognition is important to prevent complications.

**Case Presentation:**

A preterm infant born at 31 + 1 weeks of gestation was discharged on Day 22 after an initial course complicated by respiratory distress, sepsis, jaundice, and Grade I intraventricular hemorrhage (using Papile classification). On the 26^th^ day, the infant presented with mild abdominal distension and a single episode of rectal bleeding, while vital signs and laboratory findings remained stable. Nevertheless, abdominal radiography was inconclusive. However, due to recurrent rectal bleeding, abdominal ultrasonography was performed and revealed an ileocecal intussusception with preserved vascularity. The infant was managed conservatively, and serial ultrasounds demonstrated spontaneous reduction with complete resolution of symptoms.

**Conclusion:**

Intussusception in preterm neonates may present with subtle clinical signs and can mimic NEC. When abdominal radiographs are inconclusive, ultrasonography plays a crucial role in establishing an early diagnosis and may allow successful conservative management, avoiding unnecessary surgical intervention.

## 1. Introduction

Although intussusception is the most common cause of bowel obstruction in infants, it is uncommon in neonates. It is extremely rare and unusual in preterms. Possible consequences include obstruction, ischemia, and vascular compromise.

In preterm neonates, intussusception often mimics necrotizing enterocolitis (NEC), presenting with nonspecific signs such as abdominal distension, feeding intolerance, bilious gastric residuals, and rectal bleeding. Misdiagnosis is frequent, with systematic reviews reporting an average diagnostic delay of 10 days, increasing the risk of bowel compromise [1].

Diagnosing through radiologic images is difficult. Classic “target signs” may not be present on plain abdominal radiographs, which are typically nonspecific and show ileus or dilatation. The high sensitivity of abdominal ultrasonography allows for the identification of intussuscepted bowel segments and, in certain situations, preoperative diagnosis [2, 3].

Unlike full term neonates, where pathologic lead points such as Meckel’s diverticulum or duplication cysts may be present, most preterm cases lack identifiable causes [1, 4]. The site of intussusception also differs: preterm infants more frequently develop small bowel (e.g., ileoileal) intussusception, whereas full term neonates often show ileocolic involvement [4].

In this report, we describe a rare case of ileocecal intussusception in a preterm neonate, emphasizing the diagnostic challenges and the role of ultrasonography in early detection.

## 2. Case Presentation

At 31 + 1 weeks of gestation, a 25‐year‐old mother Primigravida who had previously received antibiotics for an ear infection, vaginal discharge, and premature rupture of membrane presented to A&D with labor pain. The baby was delivered vaginally, cried immediately afterward, with a good APGAR score of 9 and 10 at 1and 5 min, and admitted to the NICU for prematurity care. While his stay in NICU developed respiratory distress, early sepsis, neonatal jaundice, and Grade 1 interventricular hemorrhage (using Papile classification), he improved with appropriate care and monitoring. At 1.84 kg, he was discharged home, exclusively breast fed and in good health on the 22nd day of life.

**FIGURE 1:**
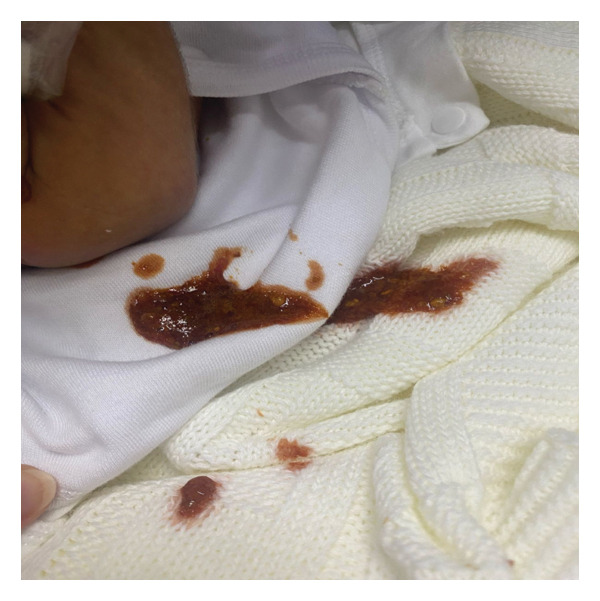
Bloody stool.

Unfortunately, he was presented to the emergency department on the 26th postnatal day (corrected gestational age 34 + 6 weeks) with one episode of rectal bleeding that was unrelated to constipation or vomiting (Figure 1). However, he was exclusively breast fed, actively crying, his vital signs were stable, and his abdomen was slightly distended, but there were no palpable masses, tenderness, or rigidity.

**FIGURE 2:**
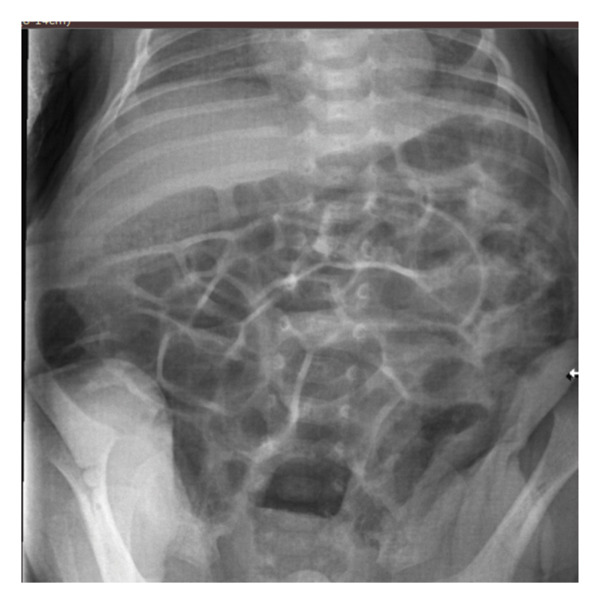
The abdominal X‐ray revealed right‐sided opacity with gas‐distended proximal and distal loops, without air–fluid levels, and gas extending to the rectum.

All laboratory investigations were normal apart from occult blood in stool. X‐ray of the abdomen revealed only opaque right‐side abdomen, the proximal and distal bowel loops have gas distention, there is no air fluid levels, and air can be traced down to the rectum (Figure 2).

**FIGURE 3:**
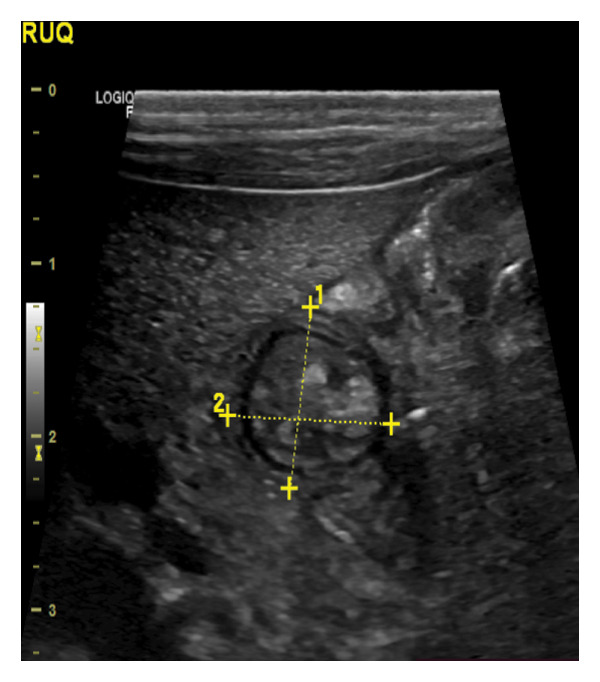
Ultrasound demonstrated focal ileocolic intussusception involving the ileum, cecum, and right colon. The intussuscepted segment measured approximately 2.5 × 1 × 1.1 cm, with the right colon up to the hepatic flexure serving as the intussusceptum. Terminal right‐colon loops showed wall thickening but preserved vascular flow. The intussusception occupied most of the right abdomen, with no free fluid seen between the intussuscipiens and intussusceptum.

After 4 hours from the first attack, there was profuse bleeding in the rectum, so differential diagnoses were listed including intussusception, NEC, sepsis‐related ileus, spontaneous intestinal perforation, milk protein intolerance, and intestinal obstruction. Given the presence of rectal bleeding with mild abdominal distension and relatively stable systemic condition, further imaging with abdominal ultrasonography was performed to clarify the diagnosis. Thus, an urgent abdominal U/S was done and showed ileocolic intussusception involving ileum, cecum, and right colon; thickened but well‐perfused bowel; and no free fluid (Figure 3).

The intravenous piperacillin/tazobactam antibiotic was started when the baby was admitted under the pediatric surgery team, but it was stopped 2 days after the blood culture was released negatively. The abdominal X‐ray was repeated on the fourth day of admission, showing fixed dilated bowel loops that progressed to the rectum. The abdominal ultrasound, which was repeated on the second and fourth days of admission, was normal. Initially, the baby was kept nil by os (NPO) but then resumed breast feeding only. On the fourth day of admission, the baby’s abdominal circumference grew from 27 to 31 cm before decreasing to 26 cm (from admission till Day 4, it was measured twice daily at 11 morning and evening). The abdominal X‐ray revealed dilated bowel loops, and eventually, the baby passed stool without blood. Thus, it was resolved and handled cautiously, with conservative management only and no surgical intervention.

The patient was managed conservatively because the initial abdominal ultrasound demonstrated intussusception; however, upon transfer to the pediatric surgery unit, a repeat abdominal ultrasound performed approximately 8 hours later showed spontaneous resolution of the intussusception. As the patient remained clinically stable with no signs of bowel obstruction, ischemia, or perforation, surgical or radiologic reduction was not indicated. Therefore, conservative management with close clinical monitoring was continued.

## 3. Discussion

Although intussusception is the most frequent cause of intestinal obstruction in infants, it is incredibly uncommon in neonates, especially in premature infants. It accounts for 0.3% of all cases of intussusception and as little as 3% of neonatal intestinal obstruction [1].

According to a systematic literature review, few cases of preterm neonates have been reported. This indicates that the pathophysiology and etiology of intussusception in these infants are unknown, and it differs significantly from preterm to full‐term neonates [5]. Some cases reported certain causes of intussusception such as hypoxia or hypoperfusion, prematurity and dysmotility [6], or due to CMV as in other reports [7]. However, in our case, we believe it is caused either by prematurity or due to idiopathic factors, resembling other cases [6, 7].

Preterm infants with intussusception have a higher risk of intestinal necrosis and subsequent perforation because the classical presentation of intussusception can only be caused by bloody diarrhea as in this case, which mimics NEC and can delay diagnosis for an average of 9.5 ± 7.3 days in most studies [1].

In contrast to NEC, where abdominal manifestations occur concurrently with the worsening of the overall condition, infants with intussusception typically have symptoms that are specifically restricted to the abdomen and the overall state does not worsen unless there is a bowel perforation [1]. In this case, the patient remained clinically stable without abdominal distension, feeding intolerance, systemic instability, or radiographic findings suggestive of NEC. Early abdominal ultrasonography was performed due to the presence of recurrent bloody stools in a high‐risk premature neonate, despite the initially mild presentation, to promptly exclude surgical causes and support early diagnosis.

Serial abdominal sonograms can be used to diagnose neonatal intussusception early [8]. This emphasizes how crucial ultrasound is when a newborn has bloody diarrhea [9].

Our case serves as an example of how abdominal ultrasonography can be used to establish an early diagnosis of intussusception in this specific group of newborns and to differentiate surgical bowel pathologies in premature neonates. Given the risks for these infants, it is important to diagnose this serious surgical issue as soon as possible to avoid a delay between the onset of the illness and the final course of treatment [5].

Neonatal intussusception does not have any conventional radiological markers. The most common imaging findings in preterm infants with intussusception are signs of ileus, such as bowel loop dilatation and occasionally gas–fluid levels. In contrast, the hallmark of NEC is pneumatosis with widespread bowel distension. However, as in our case, the X‐ray was essentially unremarkable [3].

This case adds to the limited literature demonstrating that selected preterm neonates with ileocolic intussusception may be managed conservatively under close monitoring. Moreover, educating caregivers about the importance of promptly seeking medical evaluation when concerning symptoms, particularly bloody stools, occur after the discharge is essential.

## 4. Conclusion/Learning Points

• Intussusception in preterm neonates is rare and can mimic NEC.
• Isolated rectal bleeding with stable vitals should raise suspicion.
• Radiographs may be nondiagnostic; ultrasound is essential for early detection.
• Early imaging can prevent delay and allow conservative management.
• Families of premature and very low birth weight infants should be counseled to seek immediate medical attention if alarming gastrointestinal symptoms, including bloody stools, develop after discharge.

## Funding

The authors received no financial support for the research, authorship, and/or publication of this article.

## Consent

Written informed consent was obtained from the infant’s parents for the publication of this case report and associated images.

## Conflicts of Interest

The authors declare no conflicts of interest.

## Data Availability

All data supporting the findings of this case report are included within the article. No additional datasets were generated or analyzed during the current study.
